# Sleep patterns in adults and children with less common forms of diabetes

**DOI:** 10.3389/fendo.2025.1388995

**Published:** 2025-10-13

**Authors:** Marilyn Arosemena, Maria V. Salguero, Siri Atma W. Greeley, Rochelle N. Naylor, Esra Tasali, Louis H. Philipson

**Affiliations:** ^1^ Texas Diabetes Institute - University Health, University of Texas Health San Antonio, San Antonio, TX, United States; ^2^ Universidad Espíritu Santo, Samborondón, Ecuador; ^3^ Pediatric Endocrinology, Advocate Christ Medical Center, Oak Lawn, IL, United States; ^4^ Department of Pediatrics, The University of Chicago, Chicago, IL, United States; ^5^ Department of Medicine, The University of Chicago, Chicago, IL, United States

**Keywords:** type 1 diabetes mellitus, monogenic diabetes mellitus, sleep, obstructive sleep apnea, MODY (mature onset diabetes of the young)

## Abstract

**Objective:**

To review the current evidence on sleep patterns in relation to glucose control in adults and children with type 1 diabetes (T1DM) and monogenic diabetes.

**Methods:**

We searched for the literature pertaining to T1DM and monogenic diabetes with reported sleep patterns, along with glycemic control, in PubMed. This review aimed to examine the current evidence on the relationship between sleep patterns and diabetes management and possible mediating mechanisms for this relationship in adults and children with T1DM and monogenic diabetes. We reviewed articles published from inception until 2023.

**Results:**

Twenty-five clinical studies met the eligibility criteria and were included. Children with T1DM with higher sleep variability had higher glucose levels, and those with higher glucose variability had more sleep disruptions. Comparing children with suboptimal [hemoglobin A1c (HbA1c) ≥ 7.5%] and optimal glucose control, those with suboptimal control had shorter sleep duration. There was no higher prevalence of obstructive sleep apnea (OSA) in children with T1DM compared to controls, but in T1DM, those who had OSA had higher glucose levels. Adults with T1DM had a high prevalence of poor sleep quality and were also sleeping less than the recommended hours for their age. Poor sleep quality and short sleep duration correlated with higher glycemic variability. First-generation automated insulin delivery systems did not improve sleep patterns in T1DM, but other strategies, including coaching and counseling, proved to be effective. Monogenic diabetes data also suggest poor sleep quality, short sleep duration, and high rates of sleep apnea.

**Conclusion:**

T1DM subjects seem to have worse sleep patterns, especially those with suboptimal glucose control. Monogenic diabetes data are limited, but they also suggest poor sleep patterns. Rigorous interventional studies are needed to further elucidate the sleep–diabetes relationship. Future research could provide insights into strategies that could effectively improve sleep in people living with diabetes.

## Introduction

Diabetes mellitus is a group of metabolic diseases characterized by hyperglycemia resulting from defects in insulin action, insulin secretion, or both. The goal of classifying the different types of diabetes is to better understand the cause, natural history, genetics, heritability, clinical phenotype, and optimum therapies (1).

The forms of diabetes known today include type 1 diabetes (T1DM), type 2 diabetes (T2DM), monogenic diabetes [which includes maturity-onset diabetes of the young (MODY), neonatal, mitochondrial, and syndromic], drug-induced diabetes, and other disease-associated diabetes. A unifying characteristic of diabetes is hyperglycemia, but the subtypes of diabetes differ in their etiology, natural history, associated conditions, and treatment. T2DM classically is associated with high rates of obesity and insulin resistance. T1DM is primarily an autoimmune disease seen in younger and leaner patients, but may occur at any age and any body type. Transcription factor and glucokinase mutations resulting in monogenic diabetes have a genetic background without autoimmunity and with rates of obesity and insulin resistance at or below background population levels. Other disease-associated diabetes and drug-induced types may have overlapping phenotypes in some cases, with pathognomonic features.

Sleep health has several indicators, such as sleep duration, sleep quality, and sleep timing, all of which are influenced by individual, social, and environmental demands. Sleep health is strongly linked to physical and mental well-being (2). The American Diabetes Association’s Standards of Medical Care have recommended assessment of sleep patterns as part of the comprehensive diabetes medical evaluation, given accumulated evidence highlighting the importance of sleep in glucose regulation. Meta-analyses from 15 studies suggest that both sleep duration and quality are associated with hemoglobin A1c (HbA1c) in people with T2DM (3). Subjects with type 2 diabetes additionally have higher rates of poor sleep quality, shorter sleep duration, and increased sleep apnea compared to controls (4).

Despite strong evidence linking sleep disturbances and diabetes (4, 5), previous reviews mainly focused on patients with T2DM, which often involve confounding from obesity and insulin resistance, as well as associated features of the metabolic syndrome (e.g., hypertension and hyperlipidemia). Our aim was to review the current evidence on the relationship between sleep patterns and diabetes management and possible mediating mechanisms for this relationship in adults and children with T1DM and monogenic diabetes.

## Methods

**Figure 1:**
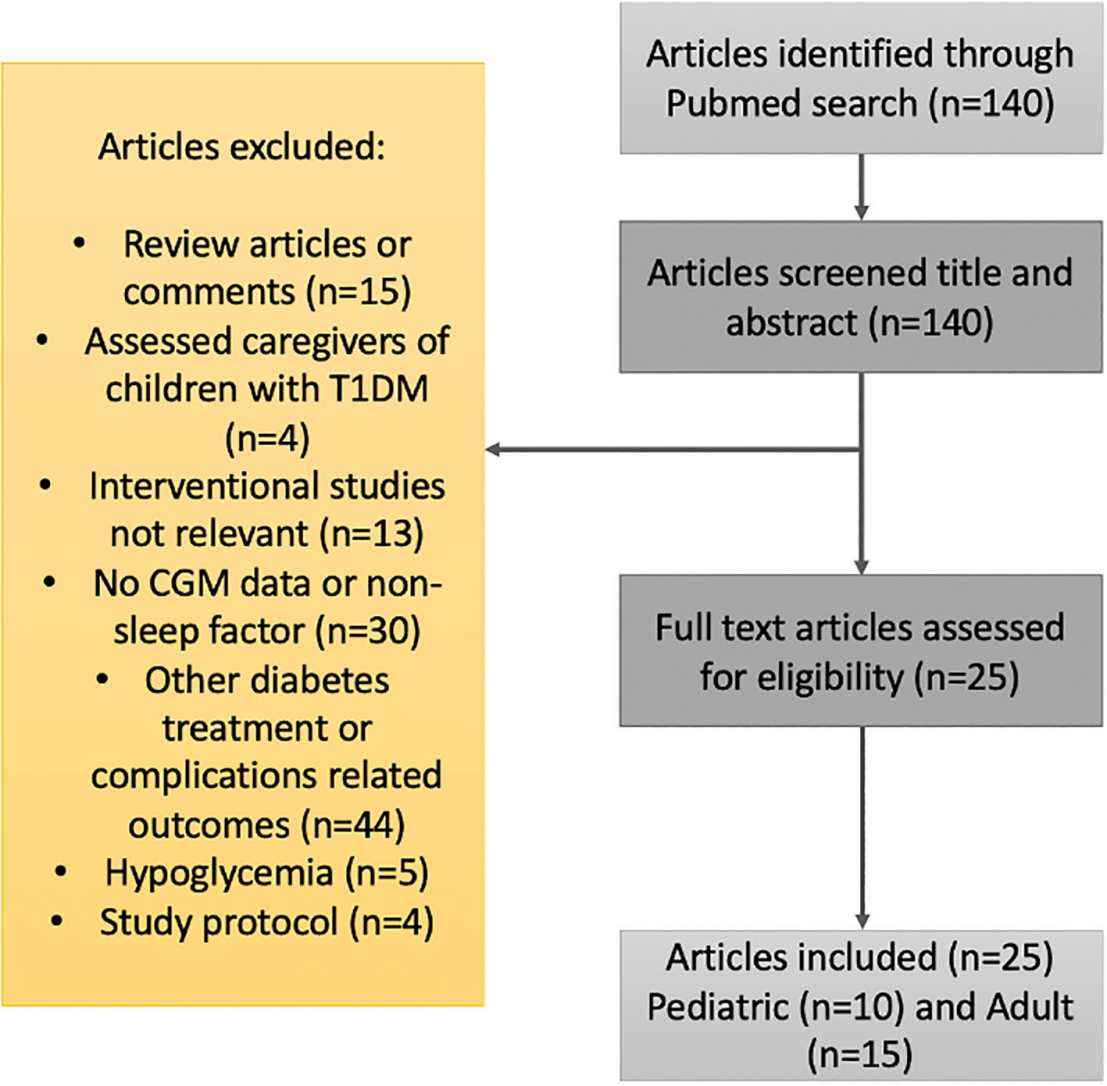
Flow diagram for the literature search and filtering of results for the review of the relationship between sleep patterns and type 1 diabetes.

Studies published in English were searched in PubMed since their inception until April 2023. For T1DM, the search terms were “type 1 diabetes”, “sleep”, “insomnia”, “apnea”, and “continuous glucose monitoring”. The search terms were employed in variable order and combinations. Inclusion criteria were studies that reported sleep characteristics with subjective and/or objective measures, along with glycemic control using continuous glucose monitoring (CGM). Exclusion criteria were reviewed articles or comments, assessment of caregivers of children with type 1 diabetes, absent sleep or CGM data, study protocols, and other diabetes treatment- or complication-related outcomes. See **Figure 1** for further details. A total of 25 clinical studies met the eligibility criteria and were included.

In children, in some circumstances, sleep duration was measured using a home sleep study or actigraphy; the majority of those studies (three out of five) used validated devices (6–8). Follow-up rate was 83%–100% in the adult studies and 52%–100% in the child studies. Although there is always risk for bias in observational studies, in the studies analyzed, the main bias we suspect may have occurred is an “observer bias” or “Hawthorne effect”. As subjects know they are being evaluated using questionnaires, wrist actigraphy, or a sleep study, they may have changed their behavior while being studied.

For monogenic diabetes, search terms included “monogenic diabetes”, “maturity-onset diabetes of the young”, “atypical diabetes”, “sleep”, “insomnia”, and “apnea”. We included studies that reported sleep characteristics with subjective and/or objective measures along with glycemic control [CGM, HbA1c, or self-monitoring of blood glucose (SMBG)]. As there were no studies including CGM or SMBG and sleep in monogenic diabetes, we included the only available three studies investigating sleep in monogenic diabetes.

### Screening for sleep disturbances

In the clinical and research settings, there are available tools to screen for sleep disorders. At a subjective level, screening can be accomplished using sleep questionnaires, and at an objective level, screening can be accomplished using in-lab or ambulatory sleep testing (9, 10). For sleep assessment methodologies, see **Table 1**. For sleep variable definitions, see **Table 2**.

**Table 1:** Sleep assessment methods.

Sleep assessment methods		
Method	Definition and methodology	Measures
Subjective methods		
Sleep diary	Subjective reports that allow patients to self-assess their sleep. Sleep diaries are filled in over a period of time (usually 1 or 2 weeks).	Sleep schedule, night waking, and related topics
Pittsburgh Sleep Quality Index	Self-rated questionnaire with 19 individual items that generate 7 component scores. The sum of scores gives a global score. Good sleep quality has a PSQI global score of ≤5, and poor sleep quality has a PSQI > 5.	Sleep quality and patterns of sleep
Epworth Sleepiness Scale	Self-administered questionnaire with 8 questions. Respondents are asked to rate their usual chances of falling asleep while engaged in eight different activities. The higher the score, the higher the daytime sleepiness.	Level of daytime sleepiness. Average sleep propensity in daily life
Berlin Questionnaire	The questionnaire consists of 3 categories related to the risk of having sleep apnea. High risk: if there are 2 or more categories where the score is positive. Low risk: if there is only 1 or no categories where the score is positive.	Risk of sleep apnea
STOP-BANG Questionnaire	Self-administered questionnaire to screen for sleep apnea. High risk of OSA: yes 5–8 Intermediate risk of OSA: yes 3–4 Low risk of OSA: yes 0–2	Risk of sleep apnea
Insomnia Severity Index (ISI)	Screening tool for insomnia with seven-item questionnaire asks respondents to rate the nature and symptoms of their sleep problems. Scores: 0–7: No clinically significant insomnia 8–14: Subthreshold insomnia 15–21: Clinical insomnia (moderate severity) 22–28: Clinical insomnia (severe)	Nature, severity, and impact of insomnia. Treatment response in adults
Morningness–Eveningness Questionnaire	Self-assessment questionnaire to determine morningness–eveningness in human circadian rhythms.	Chronotype
Objective methods		
Wrist actigraphy monitoring	Monitor that detects movement via accelerometers and has a built-in event marker. Patients press the event-marker button when they go to bed to sleep each night and when they get out of bed each morning. Sleep is then automatically scored using Actiware software, an actigraphy-based sleep-scoring program using validated algorithms.	Sleep–wake patterns
Polysomnography	The gold standard of sleep assessment. Polysomnography is based on laboratory or ambulatory monitoring that usually includes electric brain activity, muscle activation, eye movement, breathing efforts and flow, oxygen saturation sensors, and video recording.	Sleep architecture, sleep-disordered breathing, periodic movements, parasomnia, narcolepsy, REM sleep disorders, and insomnia, among others
Home Sleep Apnea Test (validated portable sleep apnea device)	A non-invasive home care device for use with patients suspected of having sleep-related breathing disorders. Measures up to 7 channels (Peripheral Arterial Tone (PAT) signal, heart rate, oximetry, actigraphy, body position, snoring, and chest motion).	Diagnosis of sleep apnea
Multiple sleep latency test	It measures how quickly a patient falls asleep during the day in a quiet environment. The standard procedure often includes EEG, EOG, EMG, and EKG.	Idiopathic hypersomnia and narcolepsy
Videosomnography	Video recordings of sleep can be conducted in the natural environment.	Sleep patterns, parental interventions, and child’s behavior during nighttime. Screen for sleep disorders: sleep apnea, night terrors, and rhythmic behaviors

PSQI, Pittsburgh Sleep Quality Index; OSA, obstructive sleep apnea; EEG, electroencephalogram; EOG, electrooculogram; EMG, electromyography; EKG, electrocardiogram.

**Table 2:** Sleep dictionary.

Sleep variable	Definition
Sleep pattern	A person’s schedule of bedtime and wake-up time, as well as nap behavior. Sleep patterns may also include time and duration of sleep interruptions.
Sleep duration	Total amount of sleep obtained, either during the nocturnal sleep episode or across the 24-hour period.
Sleep latency	Length of time, in minutes, it takes to transition from wake to sleep.
Sleep efficiency	Ratio of total sleep time to time in bed.
Wake after sleep onset	Amount of time, in minutes, spent awake after sleep has been initiated and before final awakening.
Sleep fragmentation	Interruption of sleep that involves arousals or awakenings.
Sleep onset	Falling asleep or initiating a sleep period.
Arousal	An abrupt change from a deeper to a lighter stage of sleep or from sleeping toward waking up.
Sleep variability	The standard deviation of objective sleep measures.
Sleep quality	An individual’s satisfaction with their sleep integrates aspects of sleep initiation, sleep maintenance, sleep quantity, and feeling refreshed upon awakening.
Sleep disorders	Involve problems with the quality, timing, and amount of sleep, which result in daytime distress and impairment in functioning.
Insomnia	A sleep disorder in which a person cannot fall asleep or sleep as long as they want to, even though they have the opportunity to sleep.
Sleep apnea	A type of sleep disorder marked by disordered or abnormal breathing. The two main types are obstructive sleep apnea and central sleep apnea.

Definitions obtained from the National Sleep Foundation.

## Results

### Type 1 diabetes in children

#### Glycemic control and sleep patterns

Observational studies in children have found high Pittsburgh Sleep Quality Index (PSQI) scores that are at or above the clinical cutoff for poor sleep quality (6, 7). Children who report poor sleep also report high diabetes-specific stress, poor diabetes self-management, and high state anxiety (7). A case–control study of 154 subjects, which investigated three pediatric populations (children, adolescents, and young adults), showed that young adults had higher PSQI scores than controls, while children and adolescents were no different than controls. No significant correlations were found between the self-reported sleep quality assessed using PSQI score and diabetes duration, HbA1c, the mode of therapy, the mode of glucose monitoring, or the rate of nocturnal hypo- or hyperglycemic episodes (11) (**Table 3**).

**Table 3:** Sleep patterns and glucose control in children with type 1 diabetes.

Reference, year, country	Sample (n)	Study design	Study population*	Control group	Objective sleep assessment	Subjective sleep assessment	Diabetes assessment	Main findings
Rechenberg and colleagues, 2020, USA (7)	40	Cross-sectional study	Children, age 13.4 years, 60% female, HbA1c 8.2%	No	Actigraphy (3–7 days)	Sleep diaries, PSQI, Adolescent Sleep Habits Survey	Continuous glucose monitoring (3–7 days)	Greater sleep variability was associated with higher mean glucose and higher high blood glucose index.
Sinisterra and colleagues, 2020, USA (51)	46	Cross-sectional study	Children, age 4.7 years, male 63.1%, HbA1c 7.8%	No	Actigraphy (5 days)	Sleep diary, health-related quality of life	Continuous glucose monitoring (5 days)	Average sleep time was on the lower end of the 10–13 hours recommended for this age range.
Griggs and colleagues, 2020, USA (6)	38	Cross-sectional study	Adolescent, age 13.4 years, 37.8% male, HbA1c 8.2%	No	Actigraphy (3–7 days)	Sleep diary and PSQI	Continuous glucose monitoring (3–7 days)	Greater glucose variability was associated with more awakenings, fragmentation, earlier wake time, longer WASO, and more time spent in hypoglycemia.
Perfect and colleagues, 2012, USA (16)	50	Matched case–control (age, BMI, and sex)	Youth, age 13.4 years, 58% male, HbA1c 9%, BMI Z score 65.48% ± 26.12	Yes	Single home-based polysomnography, actigraphy (5 days)	Sleep questionnaire (School Sleep Habits Survey)	Continuous glucose monitoring (5 days)	Participants with an AHI ≥ 1.5 had higher glucose levels. Sleepiness and/or poor sleep habits correlated with reduced quality of life.
Macaulay and colleagues, 2020, New Zealand (8)	82	Matched case–control (age and sex)	Children and adolescents, age 11.7 years, 53.6% male, HbA1c 8.3%, BMI Z score 0.98 (0.88)	Yes	Single-night home sleep study actigraphy (7 days)	Pediatric sleep questionnaire	Continuous glucose monitoring (7xdays)	T1DM participants with A1c ≥ 7.5% had significantly shorter total sleep time, and later sleep onset and offset than controls.
Salah and colleagues, 2020, Egypt (14)	30	Matched case–control (age and sex)	Adolescents, age 14.49 years, 50% male, HbA1c 10.8%, BMI 20.11 ± 3.71 kg/m^2^	Yes	Single night in lab polysomnography	Epworth Sleepiness Scale–Children–Adolescent	Continuous glucose monitoring (1 day)	Hyperglycemia correlated with number of awakenings, sleep-onset latency, and light sleep duration.
Kostkova and colleagues, 2018, Slovakia (17)	44	Case–control	Children, age 14.4 years, female 64%, HbA1c 9.6% BMI 21.1 ± 3.5 kg/m^2^	Yes	Single night in lab polysomnography	None	Continuous glucose monitoring (4 days)	T1DM children with more optimal short-term metabolic control had a significantly lower AHI compared to suboptimal short-term control.
Adler and colleagues, 2016, Israel (11)	154	Matched case–control (age)	Children, age 9.94 years, male 46%, HbA1c 8.1% in children, 7.9% in adolescents, 7.46% young adults	Yes	None	Sleep Disturbance Scale for Children, Adolescent Sleep–Wake Scale, PSQI, Epworth Sleepiness Scale	Continuous glucose monitoring (historic 1-year data)	T1DM adolescents had significantly lower scores in the snoring/breathing problem item compared with controls.
Pillar and colleagues, 2003, Israel and USA (18)	15	Matched case–control (age and BMI)	Adolescents, age 12.6 years, male 46%, HbA1c 8.5%, BMI 18.5 ± 2.7 kg/m^2^	Yes	Single night in lab polysomnography	None	Continuous glucose monitoring (1 day)	Hypoglycemia was associated with increased sleep efficiency and increased percentage of slow-wave sleep.
Bisio and colleagues, 2020, USA (15)	13	Non-randomized interventional	Young children, ages 7–10 years, female 62%, HbA1c 7.6%	No	None	PSQI	Continuous glucose monitoring (4 weeks)	Less parental awakening with automated insulin delivery. Parental improvement in PSQI. No change in children.

PSQI, Pittsburgh Sleep Quality Index; WASO, wake after sleep onset; AHI, apnea–hypopnea index; T1DM, type 1 diabetes; BMI, body mass index; HbA1c, hemoglobin A1c.

*Data show mean age or age range, mean hemoglobin A1c, and mean BMI included when reported.

Two observational studies in adolescents revealed that adolescents are not getting enough sleep. In one of the studies, the mean sleep duration was 7.3 hours (7) [recommended by age is 8–10 hours (12)], which is at the 40th percentile compared with age- and sex-based normative data for actigraphy-measured sleep duration (13). In both studies, over 79% of the cohort were sleeping less than the recommended hours for their age (6, 7). A study in children aged 2–5 years also showed similar results; their average sleep time was on the lower end of the 10–13 hours recommended for this age range (12). An observational age- and sex-matched case–control study also revealed that children with T1DM had later sleep onset and later sleep offset compared to controls (8).

Abnormal sleep patterns also have an impact on glucose control. Patients with greater sleep variability (i.e., standard deviation of total sleep time across the nights) had higher mean glucose levels and a higher high blood glucose index (7). Higher glucose variability was also associated with more sleep disruptions (i.e., more awakenings and sleep fragmentation) and poorer sleep [i.e., earlier wake time or longer wake after sleep onset (WASO)] in the same subject (6). Total sleep time measured using polysomnography and actigraphy was shorter in children with T1DM with suboptimal glycemic control (HbA1c ≥ 7.5%) compared to those with optimal glycemic control. Later sleep onset was also noted in those patients with suboptimal glycemic control compared to those with optimal glycemic control. Additionally, an increase in HbA1c was associated with a small but significant increase in sleep timing variability as measured using actigraphy (8).

Another case–control study of 30 children with T1DM and control siblings revealed that cases had lower sleep efficiency compared to age- and sex-matched controls. Nocturnal hypoglycemia positively correlated with the amount of deep sleep, while hyperglycemia correlated with higher sleep onset latency, decreased rapid eye movement (REM) sleep, and increased awakenings compared to those in controls (14).

An interventional study in 13 young children assessed parents’ and children’s sleep patterns by comparing sensor-augmented pump therapy vs. an automated insulin delivery system. In children, there was no difference in regard to sleep patterns and sleep quality, but there was improvement in glucose control. In their parents, there was a reduction in awakenings and improvement in the PSQI score, diabetes-related distress, and mood disorders (15).

#### Glycemic control and sleep disorders

Observational studies did not show that sleep disorders were more common in children with T1DM than in controls, with the limitation that there was a high frequency of sleep disorders among both cases and controls (11). An association was seen between the apnea–hypoxia index (AHI) and glucose. One case–control study of 50 subjects revealed that T1DM children who had obstructive sleep apnea (OSA) with an AHI ≥ 1.5 (normal AHI ≤ 1 in this population) had higher glucose levels (16). Another case–control study of 44 subjects showed similar findings, as T1DM subjects with more optimal short-term metabolic control (average glucose <180 mg/dL) had a significantly lower AHI and respiratory arousal index compared to children with suboptimal short-term control (17). The rate of change or rapid glucose concentrations was also shown to have an impact on sleep by increasing awakenings in children with T1DM (18). Sleepiness and/or poor sleep habits correlated with reduced quality of life, depressed mood, lower grades, and lower state standardized reading scores (16).

At a subjective level, children with T1DM compared to controls report significantly more sleepiness (60% vs. 31.7%, respectively) (8). At an objective level, the sleepiness may be related to an increased number of awakenings, a higher arousal index, periodic limb movements, and a higher AHI in cases compared to controls (8, 14).

### Type 1 diabetes in adults

#### Glycemic control and sleep patterns

Four cross-sectional studies with sample sizes ranging from 20 to 48 subjects revealed that a high proportion of T1DM subjects have poor sleep quality (19–22). For two of the studies, sleep quality was defined using a composite of objective sleep features (sleep efficiency, WASO, and number of awakenings) and revealed 66% of “poor sleep quality” (19) for one study and 77% for the other study (21). Subjectively measured sleep quality using sleep questionnaires revealed poor sleep quality ranging from 46% to 50% in T1DM subjects (20, 22) (**Table 4**).

**Table 4:** Sleep patterns and glucose control in adults with type 1 diabetes.

Reference, year, country	Sample size (n)	Study design	Study population	Control group	Objective	Subjective	Diabetes assessment	Main findings
Barone and colleagues, 2014, Brazil (24)	27	Matched case–control (age and BMI)	Young adults, age 26.3 years, 55% female, HbA1c 7.8%, BMI 23 ± 2.9 kg/m^2^	Yes	Actigraphy (10 days), single night in lab polysomnography	Sleep diary, Epworth Sleepiness Scale	Hemoglobin A1c, continuous glucose monitoring (1 day)	o No OSA diagnosis in young adults o Greater glycemic variability correlated with sleep latency and awakening index.
Griggs and colleagues, 2022, USA (25)	42	Cross-sectional study	Young adults, age 22.2 years, 32.6% male, HbA1c 7.2%	No	Actigraphy (7 days)	None	Continuous glucose monitoring (7xdays)	Higher glucose variability predicted poorer sleep within-person.
Brandt and colleagues, 2021, USA (19)	20	Cross-sectional study	Adults, age 30 years, 50% male, HbA1c 6.6%, 28.0 ± 6.9 kg/m^2^	No	Zmachine Insight+ (up to 15 days)	None	Hemoglobin A1c, continuous glucose monitoring (up to 15 days)	Poor sleep quality was significantly associated with greater glycemic variability.
Feupe and colleagues, 2013, USA (26)	17	Cross-sectional study	Adults, age 19–61 years, 58.8% male, HbA1c 7.3%	No	Wireless sleep monitor (4 days)	None	Continuous glucose monitoring (4 days)	o Mean sleep duration in the cohort was less than the recommended 7 hours. o There were no significant differences in glycemic range between sleep stages, but less time was spent in hypoglycemia during deep sleep.
Griggs and colleagues, 2021, USA (28)	46	Cross-sectional study	Young adults, age 22.3 years, 32.6% male, HbA1c mean 7.2%, BMI 27.0 ± 4.4 kg/m^2^	No	Actigraphy (6–14 days)	PSQI	Continuous glucose monitoring (6–14 days)	Higher inter-daily stability was associated with better objective sleep–wake characteristics.
Farabi and colleagues, 2018, USA (22)	20	Cross-sectional study	Young adults, age 18 to 39 years, HbA1c 6.8%–7.8%, BMI 25.8 ± 4.6 kg/m^2^	No	None	PSQI	Continuous glucose monitoring (3 days)	o 60% of the cohort slept less than the recommended 7 hours. o Majority of subjects reported poor sleep quality and short sleep duration. Shorter sleep duration had greater glycemic variability.
Martyn-Nemeth and colleagues, 2018, USA (20)	48	Cross-sectional study	Adults, age 27.0 years, 63% female, HbA1c 7.2%, BMI 27.1 ± 4.6 kg/m^2^	No	None	PSQI	Continuous glucose monitoring (3 days)	Poor sleep quality was significantly greater in nocturnal glycemic variability and fear of hypoglycemia.
Botella-Serrano and colleagues, 2023, Spain (21)	25	Cross-sectional study	Adults, age 38.3 years, 56% female, HbA1c 7.4%, BMI 24.4 ± 5.9 kg/m^2^	No	Fitbit (14 days)	PSQI	Continuous glucose monitoring (14 days)	Poor sleep quality was associated with lower time in range and greater glycemic variability.
Griggs and colleagues, 2022, USA (27)	46	Cross-sectional study	Adults, age 22.3 years, 67.4% female, HbA1c 7.2%, BMI 27.0 ± 4.4 kg/m^2^	No	Actigraphy (6–14 days)	PSQI	Continuous glucose monitoring (6–14 days)	There were no significant differences in glycemic range between sleep stages, but less time was spent in hypoglycemia during deep sleep.
Griggs and colleagues, 2022, USA (23)	75	Cross-sectional study	Adults, age 21.4 years, 74.7% female, HbA1c 6.8%, BMI 24.5 ± 4.6 kg/m^2^	No	None	Berlin Questionnaire, Epworth Sleepiness Scale, MEQ, PSQI	Continuous glucose monitoring**	Sleep Health Composite score was associated with higher achievement of glycemic targets.
Basille and colleagues, 2022, France (33)	46	Cross-sectional study	Adults, age 42 years, 54.3% male, HbA1c 7.6%, BMI 24.8 ± 4.1 kg/m^2^	No	Single night in lab polysomnography	None	Continuous glucose monitoring (1 day)	Sleep disorder symptoms were not more frequent in patients with above-target glucose variability.
Griggs and colleagues, 2021, USA (29)	46	Cross-sectional study	Adults, age 22.3 years, 32.6% male, HbA1c 7.2%, BMI 27.0 ± 4.4 kg/m^2^	No	Actigraphy (6–14 days)	Epworth Sleepiness Scale, PSQI	Continuous glucose monitoring (6–14 days)	o 54.3% of the sample slept less than the recommended 7 hours. o Sleep variability, daytime sleepiness, and sleep fragmentation were important factors associated with greater glucose variability.
Chakrabarti and colleagues, 2022, Australia (30)	30	Randomized, cross-over	Older adults, age 67 years, 63% women, HbA1c 7.6%, BMI 27.6 ± 3.4 kg/m^2^	No	Actigraphy (823 days)	PSQI, sleep diary	Continuous glucose monitoring (805 days)	Sleep quality was worse with closed-loop therapy compared to sensor-augmented pump therapy. Pittsburgh Sleep Quality Index did not differ with either therapy.
Bisio and colleagues, 2022, USA (31)	15	Non-randomized interventional	Older adults, age 68.7 years, 60% male, HbA1c 7%	No	Actigraphy (56 days)	PSQI	Continuous glucose monitoring (56 days)	Sleep parameters were no different between sensor-augmented therapy and AID system, except for short sleepers who had a longer sleep duration on AID.
Martyn-Nemeth and colleagues, 2022, USA (32)	14	Randomized controlled trial	Adults, age 29.7 years, 64% female, HbA1c 6.8%, BMI 26.7 ± 7.3 kg/m^2^	Yes	Actigraphy (14 days)	Epworth Sleepiness Scale	Continuous glucose monitoring (7 days)	Patients in the technology-assisted behavioral sleep intervention demonstrated an improvement in sleep regularity, reduced glycemic variability, and improved time-in-range vs. controls.

BMI, body mass index; OSA, obstructive sleep apnea; PSQI, Pittsburgh Sleep Quality Index; MEQ, Morningness–Eveningness Questionnaire; AID, automated insulin delivery; CGM, continuous glucose monitoring.

*Data show mean age or age range, mean hemoglobin A1c, and mean BMI included when reported.

**CGM days not reported.

When analyzing sleep quality with glycemia, poor sleep quality was significantly associated with greater glycemic variability after accounting for age, sex, and body mass index (BMI). Those participants with poor sleep quality had greater nocturnal glycemic variability than those with good sleep (19–21). Higher glucose levels and lower time in range were associated with poorer sleep quality (21, 22).

A composite of sleep health score (using satisfaction, alertness, timing, and efficiency) in a cross-sectional study of 75 young adults showed that better sleep health was significantly associated with higher achievement of glycemic targets (time in range and J index); however, these associations did not persist after considering covariates (T1DM duration, race, the mode of insulin delivery, and sleep apnea risk) (23).

A case–control study matched by age and BMI in 27 young adults found that neither sleep quality nor sleep duration correlated with glycemia or glycemic variability. However, in this study, sleep quality was measured differently using a visual analogue scale. Individuals with diabetes in this study presented more pronounced sleep extension from weekdays to weekends compared to controls. Glycemic variability did correlate with sleep latency and full awakening index (24).

A cross-sectional study in 42 young adults who underwent actigraphy and CGM revealed that higher sleep efficiency predicted more time in range and less time in hyperglycemia. More awakenings predicted higher glucose variability, a higher high blood glucose risk score, and more time spent in hyperglycemia between subjects. Another finding was that a higher high blood glucose index risk score and more time spent in severe hyperglycemia were associated with a longer WASO between subjects. Additionally, more time spent in severe hyperglycemia was associated with a higher sleep fragmentation index between subjects (25).

In regard to sleep duration, three cross-sectional studies with sample sizes ranging from 17 to 46 showed that subjects with T1DM are short sleepers (22, 26, 27). In one of the studies, the mean sleep duration was slightly less than 6 hours (26) [which is at the 15–25th percentile compared with age- and sex-based normative data for actigraphy-measured sleep duration (13)], and the other two studies reported that the majority of their cohort [54.3% (27) and 60% (22)] slept less than the recommended 7–9 hours. The subjects with shorter sleep duration had greater glycemic variability (22). Another cross-sectional study of 25 subjects showed a mean sleep duration of 7.15 hours (26) (at the 55th percentile compared with age- and sex-based normative data for actigraphy-measured sleep duration ^12^), higher than those reported above, but in the lower end of what is recommended for their age (12).

When investigating circadian alignment in a cross-sectional study of 46 young adults, patients with T1DM with robust and stronger rhythm (higher interdaily stability) had longer total sleep time and less self-reported daytime sleepiness, better executive function, and less hyperglycemia risk, but more time spent in hypoglycemia and greater hypoglycemia risk. Higher hypoglycemia risk was no longer significant when diabetes duration was added to the model. ^12^ In a cross-sectional study of 46 adults, poorer objective sleep–wake characteristics (longer sleep onset latency and poorer sleep efficiency) were found to be associated with higher diabetes emotional distress (27).

A cross-sectional study of 46 subjects also showed that a higher sleep fragmentation index was associated with greater glucose variability after controlling for T1DM duration and accounting for higher daytime sleepiness. Additionally, greater sleep variability was directly associated with greater glucose variability; however, this association was no longer significant when accounting for daytime sleepiness and controlling for T1DM duration (29).

Two interventional studies in older adults assessed sleep patterns and quality by comparing sensor-augmented pump therapy vs. an automated insulin delivery system. None of them found a difference in sleep quality or sleep patterns (30, 31). In one of the studies, using an automated insulin delivery system led to worse sleep quality; however, this was a first-generation closed-loop system in which patients experienced 30% more alarms compared to sensor-augmented therapy (30).

Another intervention to improve sleep health was evaluated through a randomized controlled trial in 14 adults with T1DM. A technology-assisted sleep intervention (an 8-week remotely delivered program that entailed the following: weekly emailed didactic sleep content, weekly 5–10-minute telephone coaching, and a wearable sleep tracker and smartphone application with interactive feedback and tools) demonstrated an improvement in sleep regularity, reduced glycemic variability, and improved glucose time in range compared to controls (32).

#### Glycemic control and sleep disorders

Not all studies have found a clear relationship between sleep disorders and T1DM. A case–control study in 27 T1DM young adults matched by age and BMI revealed that none of them had OSA measured using polysomnography. There was also a surprisingly negative correlation between the mean glycemia and the apnea–hypopnea index, given the established association in T2DM. Despite no detectable OSA, T1DM subjects who had the highest glycemic variability had a significantly higher awakening index (24). The same study also measured sleepiness by utilizing the Epworth Sleepiness Scale (ESS) and revealed that T1DM subjects had higher scores (more sleepiness) compared to controls (24). Similarly, a cross-sectional study of 17 subjects who utilized the same questionnaire revealed that 29% of the sample had a high ESS score (≥10), which is interpreted as daytime sleepiness (26).

A cross-sectional study of 46 older adults who underwent polysomnography and CGM showed that 37% of patients had sleep-disordered breathing (SDB; mild SDB, n = 9; moderate-to-severe SDB, n = 8). Moderate-to-severe SDB was associated with a higher BMI and a longer diabetes duration but not with above-target glucose variability or more sleep disorder symptoms. However, these findings were based on only one night of CGM, which limited the opportunity to detect possible differences (33).

### Sleep in monogenic diabetes

One study investigating sleep quality in adult MODY participants (pathogenic variants in *GCK*, *HNF4A*, *HNF1A*, and *HNF1B*) (n = 24, mean age 46.0 years, 79% women, BMI 24.7 kg/m^2^) who underwent actigraphy and answered sleep questionnaires revealed that 88% participants had poor sleep quality measured using PSQI. The mean global score was 8.8 ± 3.6. Insomnia (including subthreshold and clinical insomnia) was reported in 71% of them (34). The study also showed that 54% had sleep duration less than the recommended minimum of 7 hours. Moreover, transcription factor-related MODY (HNF4A-, HNF1A-, and HNF1B-MODY) displayed increased night-to-night variability in sleep patterns compared to GCK-MODY (34). OSA was reported at 58% (64% mild, 22% moderate, and 14% severe), measured using a home sleep monitor. OSA rate was not different in GCK-MODY vs. TF-MODY. The mean HbA1c for both groups was not significantly different (6.3% for GCK and TF-MODY) (34).

A pediatric population of 13 neonatal diabetes subjects due to a *KCNJ11* mutation revealed higher rates of sleep difficulties compared to sibling controls (35). In another study where sleep was objectively assessed using wrist actigraphy and sleep questionnaires, KCNJ11-neonatal diabetes subjects showed increased sleep duration and WASO night-to-night variability compared to unaffected siblings. Patients with neonatal diabetes had poorer sleep behaviors compared to unaffected siblings (36).

## Discussion

### Bidirectional relationship between sleep and glycemia: putative mechanisms of disrupted sleep in diabetes

**Figure 2:**
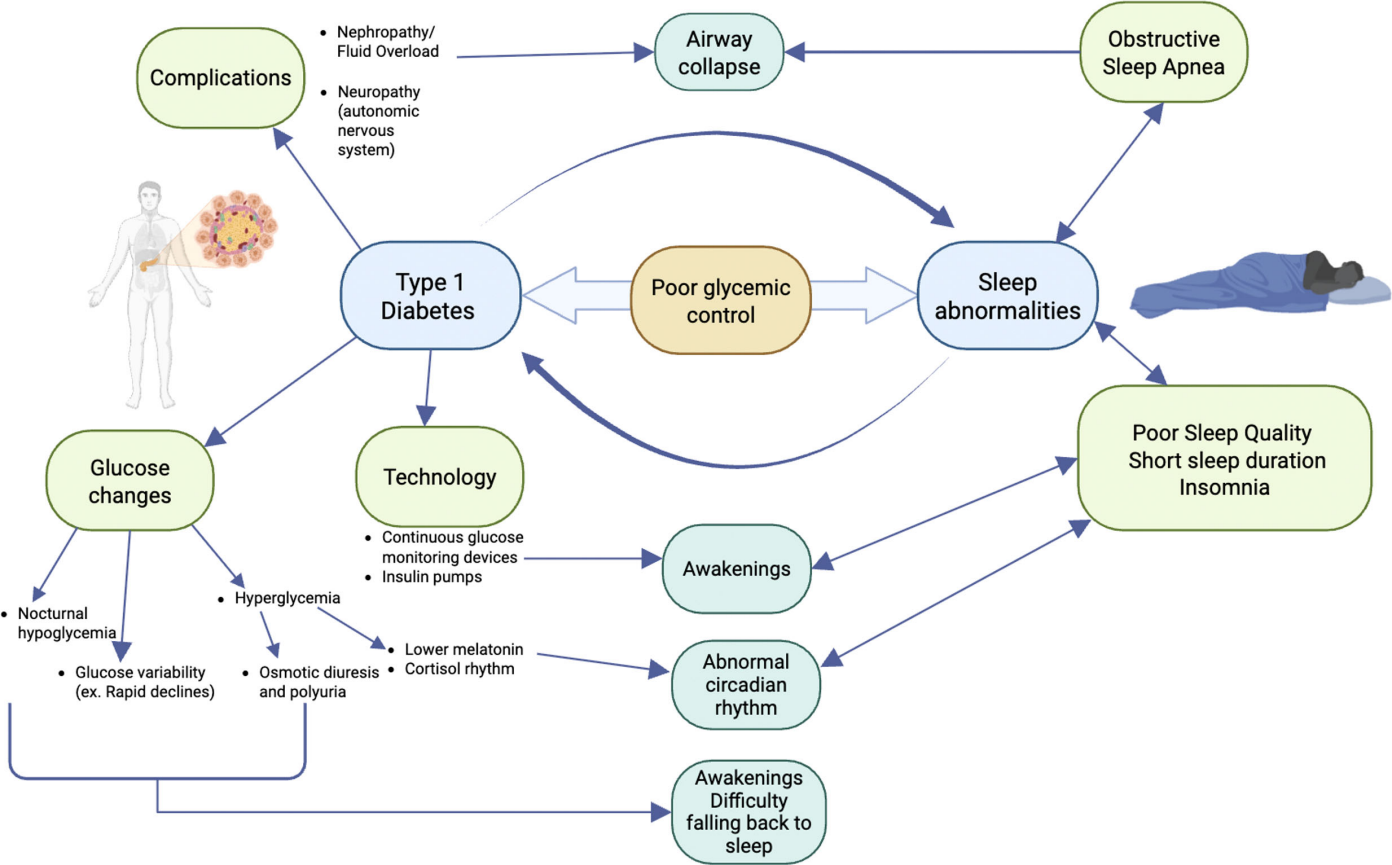
Potential mechanisms for sleep disruption in type 1 diabetes. Nephropathy with fluid overload and/or autonomic nervous system neuropathy can lead to airway collapse and obstructive sleep apnea. Glucose excursions (hypoglycemia, hyperglycemia, or high glycemic variability) can impact sleep architecture by causing awakenings and difficulty falling back to sleep or an abnormal circadian rhythm. Technology, including continuous glucose monitoring devices and/or insulin pumps, has alarms that can lead to awakenings. Sleep abnormalities, including poor sleep quality, short sleep duration, obstructive sleep apnea, and insomnia, can decrease insulin sensitivity and response; therefore, glucose control becomes more difficult.

There is growing evidence revealing how sleep and diabetes impact one another (**Figure 2**). Glucose excursions (hypo- and hyperglycemia) can impact sleep architecture (18, 21, 22). Cross-sectional studies surveying large sample sizes from different countries have shown that nocturnal hypoglycemia, even when it is not severe, correlates with patients having difficulty falling back to sleep (37, 38). Studies in children have shown that glucose variability and hyperglycemia provoke awakenings and disrupt sleep (6, 14). An expert review also hypothesized that hyperglycemia causes awakenings and disrupts sleep, as it induces osmotic diuresis, resulting in polyuria and nocturia (39). One study in T1DM showed that patients with a glucose level above 154 mg/dL had lower melatonin levels, which can impact normal circadian rhythm (40).

Once sleep is disrupted, due to short sleep duration, sleep disorders (insomnia or OSA), or poor sleep quality, glucose control worsens (8, 17). The etiology of why glucose control declines in the setting of sleep abnormalities is multifactorial. From a sleep disorder perspective, OSA increases insulin resistance, which makes diabetes control more difficult (41). OSA is most common in people who are overweight or obese; however, in the studies listed, the majority of the patients were in the “normal” or “overweight” category. Additional anthropometric parameters, aside from BMI, that also correlate with the presence and severity of OSA and cardiometabolic disease include waist and neck circumference, body shape index, body adiposity index, and abdominal volume index (42, 43). Those were not measured in these studies.

There was no increased prevalence of OSA in T1DM compared to controls in one study, while another study did show a high prevalence of OSA. The discrepancy could be secondary to differences in the studied population, as the study that found a higher prevalence of OSA included predominantly older men with higher BMI and longer diabetes duration. Awakenings in these patients may not all be explained by episodes of apnea. From a sleep pattern perspective, short sleep duration and poor sleep quality lead to decreased insulin sensitivity and insulin response; therefore, glucose control becomes more difficult. This was reported in a high-quality review that included results from randomized controlled trials and epidemiological studies (44).

Technology has brought major improvements in glucose control; however, sleep has not necessarily been impacted in a positive way (45, 46). Continuous glucose monitoring devices and pump alarms may disrupt sleep by awakening patients. The use of the newest technology with hybrid closed- and semi-closed-loop systems correlates with subjective overall positive sleep quality in one pediatric study (15), while in adult studies, results were mixed, as there was little improvement for short sleepers on newer automated insulin delivery (AID) systems but worse sleep quality with older-generation automated systems due to alarms (30, 31). However, this conclusion relates to limited data in a small population with mostly first-generation systems and may change over time.

Sleep and mood disorders also have a significant impact on each other and should be taken into consideration, as they are highly prevalent in people with diabetes (47, 48). Sleepiness and/or poor sleep habits correlate with reduced quality of life, depressed mood, lower grades, and lower state standardized reading scores in children (16). One study reported that 36% of the sample screened positive for a mood disorder (21), and another study reported high depression scores (31). The rest of the studies described in this review did not screen for or report mood disorders, while others excluded patients with major psychiatric comorbidities as part of the study design (25).

The limitations of this literature review include studies with small sample sizes, short sleep and glycemic follow-up, and the potential for the Hawthorne effect affecting results. Most studies were cross-sectional studies, which have a lower level of evidence, making it difficult to establish cause–effect relationships and are prone to bias. Another limitation is the difficulty in systematically comparing studies that use different measurement tools (subjective and objective) for evaluating sleep patterns and sleep quality, which can lead to different results. Many studies did not control for confounding factors such as depression, obesity, hypertension, alcohol consumption, and obstructive sleep apnea. Furthermore, in both adult and pediatric studies, population characteristics differ in terms of BMI, age range, gender predominance, and glycemic control, which may also affect the results.

## Summary and future directions

There is strong evidence in T2DM that sleep characteristics can positively or negatively impact the neuroendocrine systems, while diabetes itself often leads to sleep difficulties and disturbances (49, 50). Subjects with type 2 diabetes have been more extensively studied, and it is known that they have shorter sleep duration, poorer sleep quality, and increased sleep apnea compared to controls and the rates reported in other types of diabetes. The reciprocal relationship between T1DM and sleep is not completely well understood and needs more rigorous interventional studies. When considering the available evidence with its limitations, data have shown that short sleep duration, poor sleep quality, and sleep disorders can negatively impact glycemic control (19, 21–23). Children with T1DM have higher PSQI scores, indicating poor sleep quality (6, 7). Objectively, most children with T1DM are sleeping less than the recommended hours for their age (6, 7, 51). Those patients who elicit higher sleep variability have higher glucose levels, and patients with higher glucose variability have more sleep disruptions (6–8). Studies have also revealed that in people with diabetes, those with suboptimal control have shorter sleep duration compared to those with optimal control (8). Children with T1DM who have a higher apnea–hypopnea index also have higher glucose levels, and when rapid changes in glucose levels occur, they have more awakenings (6, 16).

In adults, subjectively and objectively, most studies have shown a high percentage of poor sleep quality in people with T1DM (19–22). Poor sleep quality is related to glycemic variability; higher glycemic variability is associated with poorer sleep quality. Out-of-range glucose is also associated with poor sleep quality (19–22). Hyperglycemia also negatively impacts adult sleep patterns, causing lower sleep efficiency, more awakenings, more sleep fragmentation, and higher WASO (24, 25). Sleep duration across all studies shows that most patients with T1DM sleep less than the recommended sleep duration for their age. Those subjects who have shorter sleep duration also exhibit higher glycemic variability, and poor sleep–wake characteristics have also been shown to produce higher emotional distress (22, 26, 27).

It is unclear if the use of automated insulin delivery systems can improve sleep patterns and quality in T1DM, as the devices’ alarms alert users and can disrupt sleep. Other strategies, including coaching and counseling, have proven to be effective (30–32).

In MODY, it is not yet clear what may be driving the higher rates of OSA, insomnia, and poor sleep quality. It is also unclear what underlies the decreased sleep variability seen in GCK-MODY compared to TF-MODY (34). KCNJ11-neonatal diabetes appears also to be commonly affected by sleep disturbances, seemingly attributable to the impairment of KATP channel function in the brain; however, more extensive studies need to be performed (35).

We have made great progress in understanding that sleep disruption is common in diabetes, but also that blood glucose control appears to be worse in those with disrupted sleep. Although there are no clear mechanisms of cause and effect for sleep disturbances, possible contributors include diabetes-related complications, sleep-disrupting technology, and glycemic variability or out-of-range glycemia. Advances in technology and data science have the potential to help us better understand this relationship. Learning that individuals with certain types of diabetes are at particularly high risk for developing certain sleep disorders will improve screening and treatment strategies (52). In the coming years, there is a need for both pediatric and adult large-scale cohort studies that can evaluate longitudinally the concomitant use of actigraphy, polysomnography, and CGM with standardized sleep metrics, as well as interventional studies targeting sleep hygiene strategies to improve sleep quality in both T1DM and monogenic diabetes. This will better elucidate the relationship between sleep and diabetes in less common forms of diabetes.

## References

[1] Thomas CC Philipson LH. Update on diabetes classification. Med Clin North Am. (2015) 99:1–16. doi: 10.1016/j.mcna.2014.08.015, PMID: 25456640

[2] Buysse DJ. Sleep health: can we define it? Does it matter? Sleep. (2024) 37(1):9–17. doi: 10.5665/sleep.3298, PMID: 24470692 PMC3902880

[3] Lee SWH Ng KY Chin WK. The impact of sleep amount and sleep quality on glycemic control in type 2 diabetes: A systematic review and meta-analysis. Sleep Med Rev. (2017) 31:91–101. doi: 10.1016/j.smrv.2016.02.001, PMID: 26944909

[4] Nefs GM Bazelmans E Donga E Tack CJ de Galan BE. Sweet dreams or bitter nightmare: a narrative review of 25 years of research on the role of sleep in diabetes and the contributions of behavioural science. Diabetes Med. (2020) 37:418–26. doi: 10.1111/dme.14211, PMID: 31833083

[5] Rutters F Nefs G. Sleep and circadian rhythm disturbances in diabetes: A narrative review. Diabetes Metab Syndr Obes. (2022) 15:3627–37. doi: 10.2147/DMSO.S354026, PMID: 36439294 PMC9694979

[6] Griggs S Redeker NS Jeon S Grey M. Daily variations in sleep and glucose in adolescents with type 1 diabetes. Pediatr Diabetes. (2020) 21:1493–501. doi: 10.1111/pedi.13117, PMID: 32902901 PMC7642150

[7] Rechenberg K Griggs S Jeon S Redeker N Yaggi HK Grey M. Sleep and glycemia in youth with type 1 diabetes. J Pediatr Health Care. (2020) 34:315–24. doi: 10.1016/j.pedhc.2019.12.002, PMID: 32171612 PMC7311270

[8] Macaulay GC Galland BC Boucher SE Wiltshire EJ Haszard JJ Campbell AJ. Impact of type 1 diabetes mellitus, glucose levels, and glycemic control on sleep in children and adolescents: a case-control study. Sleep. (2020) 43:zsz226. doi: 10.1093/sleep/zsz226, PMID: 31583407

[9] Ibáñez V Silva J Cauli O. A survey on sleep questionnaires and diaries. Sleep Med. (2018) 42:90–6. doi: 10.1016/j.sleep.2017.08.026, PMID: 29458752

[10] Sadeh A. Sleep assessment methods. Monogr Soc Res Child Dev. (2015) 80:33–48. doi: 10.1111/mono.12143, PMID: 25704734

[11] Adler A Gavan MY Tauman R Phillip M Shalitin S. Do children, adolescents, and young adults with type 1 diabetes have increased prevalence of sleep disorders? Pediatr Diabetes. (2017) 18:450–8. doi: 10.1111/pedi.12419, PMID: 27488802

[12] Hirshkowitz M Whiton K Albert SM Alessi C Bruni O DonCarlos L. National Sleep Foundation’s sleep time duration recommendations: methodology and results summary. Sleep Health. (2015) 1:40–3. doi: 10.1016/j.sleh.2014.12.010, PMID: 29073412

[13] Barreira TV Schuna JM Chaput JP. NORMATIVE REFERENCE VALUES FOR ACTIGRAPHY-MEASURED TOTAL NOCTURNAL SLEEP TIME IN THE US POPULATION. Am J Epidemiol. (2022) 191:360–2. doi: 10.1093/aje/kwab258, PMID: 34668972

[14] Salah NY Abido AY Rashed HR. Relationship of glycaemic derangement using continuous glucose monitoring system with sleep pattern among children with type 1 diabetes. Diabetes Metab Res Rev. (2021) 37:e3407. doi: 10.1002/dmrr.3407, PMID: 32935448

[15] Bisio A Brown SA McFadden R Pajewski M Yu PL DeBoer M. Sleep and diabetes-specific psycho-behavioral outcomes of a new automated insulin delivery system in young children with type 1 diabetes and their parents. Pediatr Diabetes. (2021) 22:495–502. doi: 10.1111/pedi.13164, PMID: 33289242

[16] Perfect MM Patel PG Scott RE Wheeler MD Patel C Griffin K. Sleep, glucose, and daytime functioning in youth with type 1 diabetes. Sleep. (2012) 35:81–8. doi: 10.5665/sleep.1590, PMID: 22215921 PMC3242691

[17] Kostkova M Durdik P Ciljakova M Vojtkova J Sujanska A Pozorciakova K. Short-term metabolic control and sleep in children and adolescents with type 1 diabetes mellitus. J Diabetes Complications. (2018) 32:580–5. doi: 10.1016/j.jdiacomp.2018.03.010, PMID: 29709336

[18] Pillar G Schuscheim G Weiss R Malhotra A McCowen KC Shlitner A. Interactions between hypoglycemia and sleep architecture in children with type 1 diabetes mellitus. J Pediatr. (2003) 142:163–8. doi: 10.1067/mpd.2003.66, PMID: 12584538

[19] Brandt R Park M Wroblewski K Quinn L Tasali E Cinar A. Sleep quality and glycaemic variability in a real-life setting in adults with type 1 diabetes. Diabetologia. (2021) 64:2159–69. doi: 10.1007/s00125-021-05500-9, PMID: 34136937 PMC9254230

[20] Martyn-Nemeth P Phillips SA Mihailescu D Farabi SS Park C Lipton R. Poor sleep quality is associated with nocturnal glycaemic variability and fear of hypoglycaemia in adults with type 1 diabetes. J Adv Nurs. (2018) 74:2373–80. doi: 10.1111/jan.13765, PMID: 29917259 PMC7778470

[21] Botella-Serrano M Velasco JM Sánchez-Sánchez A Garnica O Hidalgo JI. Evaluating the influence of sleep quality and quantity on glycemic control in adults with type 1 diabetes. Front Endocrinol (Lausanne). (2023) 14:998881. doi: 10.3389/fendo.2023.998881, PMID: 36896174 PMC9989462

[22] Farabi SS Quinn L Phillips S Mihailescu D Park C Ali M. Endothelial dysfunction is related to glycemic variability and quality and duration of sleep in adults with type 1 diabetes. J Cardiovasc Nurs. (2018) 33:E21–5. doi: 10.1097/JCN.0000000000000485, PMID: 29629915

[23] Griggs S Pignatiello G Hickman RL. A composite measure of sleep health is associated with glycaemic target achievement in young adults with type 1 diabetes. J Sleep Res. (2022) 32(3):e13784. doi: 10.1111/jsr.13784, PMID: 36372966 PMC10176021

[24] Barone MTU Wey D Schorr F Franco DR Carra MK Lorenzi Filho G. Sleep and glycemic control in type 1 diabetes. Arch Endocrinol Metab. (2015) 59:71–8. doi: 10.1590/2359-3997000000013, PMID: 25926118

[25] Griggs S Grey M Strohl KP Crawford SL Margevicius S Kashyap SR. Variations in sleep characteristics and glucose regulation in young adults with type 1 diabetes. J Clin Endocrinol Metab. (2022) 107:e1085–95. doi: 10.1210/clinem/dgab771, PMID: 34698348 PMC8852208

[26] Feupe SF Frias PF Mednick SC McDevitt EA Heintzman ND. Nocturnal continuous glucose and sleep stage data in adults with type 1 diabetes in real-world conditions. J Diabetes Sci Technol. (2013) 7:1337–45. doi: 10.1177/193229681300700525, PMID: 24124962 PMC3876379

[27] Griggs S Grey M Ash GI Li CSR Crawford SL Hickman RL. Objective sleep-wake characteristics are associated with diabetes symptoms in young adults with type 1 diabetes. Sci Diabetes Self Manag Care. (2022) 48:149–56. doi: 10.1177/26350106221094521, PMID: 35446182 PMC9157415

[28] Griggs S Strohl KP Grey M Barbato E Margevicius S Hickman RL. Circadian characteristics of the rest-activity rhythm, executive function, and glucose fluctuations in young adults with type 1 diabetes. Chronobiol Int. (2021) 38:1477–87. doi: 10.1080/07420528.2021.1932987, PMID: 34128443 PMC8403141

[29] Griggs S Hickman RL Strohl KP Redeker NS Crawford SL Grey M. Sleep-wake characteristics, daytime sleepiness, and glycemia in young adults with type 1 diabetes. J Clin Sleep Med. (2021) 17:1865–74. doi: 10.5664/jcsm.9402, PMID: 33949941 PMC8636341

[30] Chakrabarti A Trawley S Kubilay E Mohammad Alipoor A Vogrin S Fourlanos S. Closed-loop insulin delivery effects on glycemia during sleep and sleep quality in older adults with type 1 diabetes: results from the ORACL trial. Diabetes Technol Ther. (2022) 24:666–71. doi: 10.1089/dia.2022.0110, PMID: 35575751

[31] Bisio A Gonder-Frederick L McFadden R Cherñavvsky D Voelmle M Pajewski M. The impact of a recently approved automated insulin delivery system on glycemic, sleep, and psychosocial outcomes in older adults with type 1 diabetes: A pilot study. J Diabetes Sci Technol. (2022) 16:663–9. doi: 10.1177/1932296820986879, PMID: 33451264 PMC9294584

[32] Martyn-Nemeth P Duffecy J Quinn L Steffen A Baron K Chapagai S. Sleep-opt-in: A randomized controlled pilot study to improve sleep and glycemic variability in adults with type 1 diabetes. Sci Diabetes Self Manag Care. (2023) 49:11–22. doi: 10.1177/26350106221136495, PMID: 36453165 PMC9983445

[33] Basille D Timmerman M Basille-Fantinato A Al-Salameh A Fendri S Lalau JD. Screening for sleep-disordered breathing in people with type 1 diabetes by combining polysomnography with glucose variability assessment. Diabetes Res Clin Pract. (2022) 185:109786. doi: 10.1016/j.diabres.2022.109786, PMID: 35182713

[34] Arosemena M Salguero MV Naylor RN Wroblewski K Tasali E Philipson LH. Objective and subjective sleep patterns in adults with maturity-onset diabetes of the young (MODY). Diabetes Care. (2023) 46(3):608–12. doi: 10.2337/dc22-1343, PMID: 36637968 PMC10090264

[35] Landmeier KA Lanning M Carmody D Greeley SAW Msall ME. ADHD, learning difficulties and sleep disturbances associated with KCNJ11-related neonatal diabetes. Pediatr Diabetes. (2017) 18:518–23. doi: 10.1111/pedi.12428, PMID: 27555491 PMC5720354

[36] Tian P Hendrix KR Choppara S Wroblewski K Letourneau-Freiberg LR Spruyt K. Increased Actigraphy-Based Sleep Variability and Poor Sleep Behaviors in KATPChannel-Related Neonatal Diabetes Mellitus (KATP-NDM) Individuals Compared to Unaffected Siblings. Abstracts of the 2022 Pediatric Endocrine Society (PES) Annual Meeting. Horm Res Paediatr. (2022) 95 Suppl (Hormone research in pediatrics, Karger Publishers: Basel, Switzerland). 1:1–266.

[37] Brod M Pohlman B Wolden M Christensen T. Non-severe nocturnal hypoglycemic events: experience and impacts on patient functioning and well-being. Qual Life Res. (2013) 22:997–1004. doi: 10.1007/s11136-012-0234-3, PMID: 22825805 PMC3664748

[38] Brod M Christensen T Bushnell DM. Impact of nocturnal hypoglycemic events on diabetes management, sleep quality, and next-day function: results from a four-country survey. J Med Econ. (2012) 15:77–86. doi: 10.3111/13696998.2011.624144, PMID: 22029460

[39] Farabi SS. Type 1 diabetes and sleep. Diabetes Spectr. (2016) 29:10–3. doi: 10.2337/diaspect.29.1.10, PMID: 26912959 PMC4755454

[40] Amaral FG Turati AO Barone M Scialfa JH do Carmo Buonfiglio D Peres R. Melatonin synthesis impairment as a new deleterious outcome of diabetes-derived hyperglycemia. J Pineal Res. (2014) 57:67–79. doi: 10.1111/jpi.12144, PMID: 24819547

[41] Cho Y. Early development of bidirectional associations between sleep disturbance and diabetes. Diabetes Metab J. (2020) 44:668–70. doi: 10.4093/dmj.2020.0198, PMID: 33115210 PMC7643604

[42] Balat K Pazarlı AC İnönü Köseoğlu H Yaşayancan N Demir O. Importance of anthropometric measurements to determine cardiometabolic diseases in obstructive sleep apnea syndrome. Turk Thorac J. (2021) 22:11–7. doi: 10.5152/TurkThoracJ.2020.19105, PMID: 33646098 PMC7919437

[43] Pazarlı AC. The role of anthropometric measurements in identifying cardiometabolic diseases in obstructive sleep apnea syndrome. Tuberk Toraks. (2022) 70:287–92. doi: 10.5578/tt.20229708, PMID: 36164953

[44] Spiegel K Tasali E Leproult R Van Cauter E. Effects of poor and short sleep on glucose metabolism and obesity risk. Nat Rev Endocrinol. (2009) 5:253–61. doi: 10.1038/nrendo.2009.23, PMID: 19444258 PMC4457292

[45] Shivers JP Mackowiak L Anhalt H Zisser H. Turn it off!”: diabetes device alarm fatigue considerations for the present and the future. J Diabetes Sci Technol. (2013) 7:789–94. doi: 10.1177/193229681300700324, PMID: 23759412 PMC3869147

[46] Messer LH Johnson R Driscoll KA Jones J. Best friend or spy: a qualitative meta-synthesis on the impact of continuous glucose monitoring on life with Type 1 diabetes. Diabetes Med. (2018) 35:409–18. doi: 10.1111/dme.13568, PMID: 29247556

[47] Buchberger B Huppertz H Krabbe L Lux B Mattivi JT Siafarikas A. Symptoms of depression and anxiety in youth with type 1 diabetes: A systematic review and meta-analysis. Psychoneuroendocrinology. (2016) 70:70–84. doi: 10.1016/j.psyneuen.2016.04.019, PMID: 27179232

[48] Benca RM Okawa M Uchiyama M Ozaki S Nakajima T Shibui K. Sleep and mood disorders. Sleep Med Rev. (1997) 1:45–56. doi: 10.1016/S1087-0792(97)90005-8, PMID: 15310523

[49] Huang T Lin BM Stampfer MJ Tworoger SS Hu FB Redline S. A population-based study of the bidirectional association between obstructive sleep apnea and type 2 diabetes in three prospective U.S. Cohorts. Diabetes Care. (2018) 41:2111–9. doi: 10.2337/dc18-0675, PMID: 30072403 PMC6150434

[50] Aurora RN Punjabi NM. Obstructive sleep apnoea and type 2 diabetes mellitus: a bidirectional association. Lancet Respir Med. (2013) 1:329–38. doi: 10.1016/S2213-2600(13)70039-0, PMID: 24429158

[51] Sinisterra M Hamburger S Tully C Hamburger E Jaser S Streisand R. Young children with type 1 diabetes: sleep, health-related quality of life, and continuous glucose monitor use. Diabetes Technol Ther. (2020) 22:639–42. doi: 10.1089/dia.2019.0437, PMID: 32027177 PMC7406998

[52] Griffin S. Diabetes precision medicine: plenty of potential, pitfalls and perils but not yet ready for prime time. Diabetologia. (2022) 65:1913–21. doi: 10.1007/s00125-022-05782-7, PMID: 35999379 PMC9522689

